# Molecular and pathobiological characterization of 61 *Potato mop‐top virus* full‐length cDNAs reveals great variability of the virus in the centre of potato domestication, novel genotypes and evidence for recombination

**DOI:** 10.1111/mpp.12552

**Published:** 2017-05-11

**Authors:** Pruthvi Kalyandurg, Jose Fernando Gil, Nina I. Lukhovitskaya, Betty Flores, Giovanna Müller, Carlos Chuquillanqui, Ladislao Palomino, Aderito Monjane, Ian Barker, Jan Kreuze, Eugene I. Savenkov

**Affiliations:** ^1^ Department of Plant Biology, Uppsala BioCenter SLU Swedish University of Agricultural Sciences, Linnean Center for Plant Biology Uppsala 75007 Sweden; ^2^ International Potato Center (CIP) Apartado 1558 Lima 12 Peru; ^3^ Instituto Nacional de Innovación Agraria (INIA) EEA – Andenes 04540 Cuzco Peru; ^4^ Present address: Division of Virology, Department of Pathology University of Cambridge Hills Road Cambridge CB2 0QQ UK; ^5^ Present address: Norwegian Veterinary Institute 0106 Oslo Norway; ^6^ Present address: Syngenta Foundation for Sustainable Agriculture 4002 Basel Switzerland

**Keywords:** diversifying selection, evolutionary divergence, genotype constellation, infectious full‐length cDNA clone, multipartite virus, *Potato mop‐top virus*, reassortment

## Abstract

The evolutionary divergence of *Potato mop‐top virus* (PMTV), a tri‐partite, single‐stranded RNA virus, is exceptionally low, based on the analysis of sequences obtained from isolates from Europe, Asia and North America. In general, RNA viruses exist as dynamic populations of closely related and recombinant genomes that are subjected to continuous genetic variation. The reason behind the low genetic variation of PMTV remains unclear. The question remains as to whether the low variability is a shared property of all PMTV isolates or is a result of the limited number of isolates characterized so far. We hypothesized that higher divergence of the virus might exist in the Andean regions of South America, the centre of potato domestication. Here, we report high variability of PMTV isolates collected from 12 fields in three locations in the Andean region of Peru. To evaluate PMTV genetic variation in Peru, we generated full‐length cDNA clones, which allowed reliable comparative molecular and pathobiological characterization of individual isolates. We found significant divergence of the *CP‐RT* and *8K* sequences. The *8K* cistron, which encodes a viral suppressor of RNA silencing, was found to be under diversifying selection. Phylogenetic analysis determined that, based on the *CP‐RT* sequence, all PMTV isolates could be categorized into three separate lineages (clades). Moreover, we found evidence for recombination between two clades. Using infectious cDNA clones of the representatives of these two clades, as well as reassortants for the RNA‐CP genomic component, we determined the pathobiological differences between the lineages, which we coined as S (for severe) and M (for mild) types. Interestingly, all isolates characterized previously (from Europe, Asia and North America) fall into the S‐type clade, whereas most of the Peruvian isolates belong to the M‐type. Taken together, our results support the notion of the single introduction of PMTV from the centre of potato origin to Europe, and subsequent spread of the S‐type into Asia and USA. This is also supported by the suggested novel classification of isolates based on genetic constellations.

## Introduction

RNA viruses within a host are faced with continuous changes in host gene expression and metabolism, and trigger numerous different antiviral reactions, e.g. RNA silencing. As a result, in order to cope with antiviral responses and to evade them, RNA virus genomes exhibit extremely high mutation rates owing to the lack of proofreading activity and elevated error rate (10^−4^–10^−6^ errors per nucleotide) residue of their RNA‐dependent RNA polymerases (RdRps). Surprisingly, some RNA viruses with multipartite genomes, *e.g. Potato mop‐top virus* (PMTV), show variability as low as 0%–2% depending on the isolate (Beuch *et al*., 2015; Hu *et al*., 2016; Latvala‐Kilby *et al*., 2009; Ramesh *et al*., 2014). We speculated that the high genomic stability and low sequence variation of PMTV, to preserve genome integrity, might be a result of constraints imposed by the need to transport three RNA genomic components and the mode of PMTV movement in the plant as polar virions, which require rather intricate interactions between the minor coat protein (also known as CP‐RT), viral movement protein and, possibly, certain host factors at one extremity of the virus particles, leaving little room for variation (Torrance *et al*., 2009). However, the low fidelity of replication seems to be essential for viral survival in the host ecosystem, because virus variants with atypically low mutation rates lose fitness (Coffey *et al*., 2011; Pfeiffer and Kirkegaard, 2005). The question still remains as to whether a sufficient number of PMTV isolates have been sequenced to claim low genetic variation of PMTV in general. Recently, a variety of genetically distinct PMTV variants have been identified in Colombia, suggesting that, compared with previous reports, higher PMTV diversity may be found in South America (Gil *et al*., 2016), where the potato, the main host of PMTV, is often propagated vegetatively.

The potato is the third most important food crop in the world (International Potato Center, 2014), with an annual production of more than 380 million tons [Food and Agriculture Organization (FAO), 2017]. More than one‐third of the global potato output now comes from developing countries. However, as it is a vegetatively propagated crop in these countries, potato is particularly prone to the buildup of systemic diseases, mainly caused by viruses. Considering that the potato's area of origin lies within contemporary Peru (Spooner *et al*., 2005), it is not surprising that many viruses that affect potato have been identified there, and it is in Peru where the greatest variability of viruses is found (Hooker, 1982). In the Andean region of South America, and elsewhere in the world, there has been an increase in the incidence of PMTV. PMTV was first reported in Peru in the 1970s (Salazar and Jones, 1975). A study performed at the International Potato Center (known by its Spanish acronym, CIP) indicated that all commercial cultivars tested were susceptible to Peruvian isolates of PMTV (Tenorio *et al*., 2006).

PMTV causes significant economic losses to potato production by reducing tuber quality (Santala *et al*., 2010). PMTV infection is characterized by typical ‘spraing’ symptoms in the tuber, which consist of necrotic rings and flecks in the tuber flesh (Calvert and Harrison, 1966). The occurrence of the virus has been reported from many parts of the world, including Nordic countries, the Americas and parts of Asia (Hu *et al*., 2016; Ramesh *et al*., 2014; Santala *et al*., 2010). The recent discovery of new genetic variants of the virus in USA and Colombia raises concern about the epidemiological risk this might bring to territories in which these variants are not present (Gil *et al*., 2016; Ramesh *et al*., 2014).

PMTV is the type member of the genus *Pomovirus* within the family *Virgaviridae* (Adams *et al*., 2012). Its genome consists of three single‐stranded RNA segments of plus sense polarity. The RNA‐rep segment encodes RdRp, the replicase subunits of the virus (Savenkov *et al*., 1999), whereas RNA‐CP encodes a coat protein (CP) and a minor CP (CP‐RT), which is produced by read‐through of the CP stop codon (Kashiwazaki *et al*., 1995). The read‐through domain of the CP‐RT protein is important for the transmission of PMTV, as deletions in the read‐through region result in the loss of transmissibility of the virus by its soil‐borne vector *Spongospora subterranea* (Reavy *et al*., 1998). Moreover, CP‐RT is localized to the cell periphery, probably by association with the plasma membrane and plasmodesmata (Chapman *et al*., 2008). As it is a component of virions, CP‐RT is involved in the systemic movement of virus particles through interaction with a movement protein, TGB1 (Torrance *et al*., 2009). RNA‐TGB encodes a movement module, the triple gene block (TGB) of movement proteins (Scott *et al*., 1994), and a viral suppressor of RNA silencing (VSR), the 8K protein (Lukhovitskaya *et al*., 2013). The virus genome also includes 3′‐terminal tRNA‐like structures present in all RNA segments (Savenkov *et al*., 1999). The sequences of tRNA‐like structures are identical in all three genome components, and in all PMTV isolates sequenced so far (Gil *et al*., 2016). Moreover, the sequences of the 5′‐untranslated regions (5′‐UTRs) contain some common elements among genome segments and are well conserved in all PMTV isolates (Gil *et al*., 2016; Savenkov *et al*., 1999). This incredible conservation of the PMTV 3′‐ and 5′‐terminal sequences could be an advantage in the design of specific primers to amplify complete genomic components of uncharacterized isolates of PMTV.

In this study, we analysed the complete sequences of 61 cDNA clones of Peruvian isolates of PMTV to determine the phylogeny, genotype composition and possible recombination events. The PMTV isolates worldwide were phylogenetically classified into two (RNA‐rep and RNA‐TGB segment) or three (RNA‐CP) lineages. Based on the RNA‐CP sequences, the isolates from Peru were classified into three lineages, showing the greatest variability relative to isolates from the rest of the world represented by a single lineage. Pathobiological characterization of the Peruvian isolates using infectious clones revealed two distinct strains of the virus: S (severe) and M (mild). Further reassortment experiments identified the RNA‐CP segment as being responsible for the distinction between S and M types, and the pathobiological properties of the strains were consistent with the phylogeny of the RNA‐CP segment. Both S and M strains are found in Peru, whereas, so far, only the S strain has been reported from the rest of the world, suggesting the single introduction of PMTV into Europe and/or subsequent spread to Asia and North America.

## Results

### Distribution of genetic variability within field isolates of PMTV in Peru

**Figure 1:**
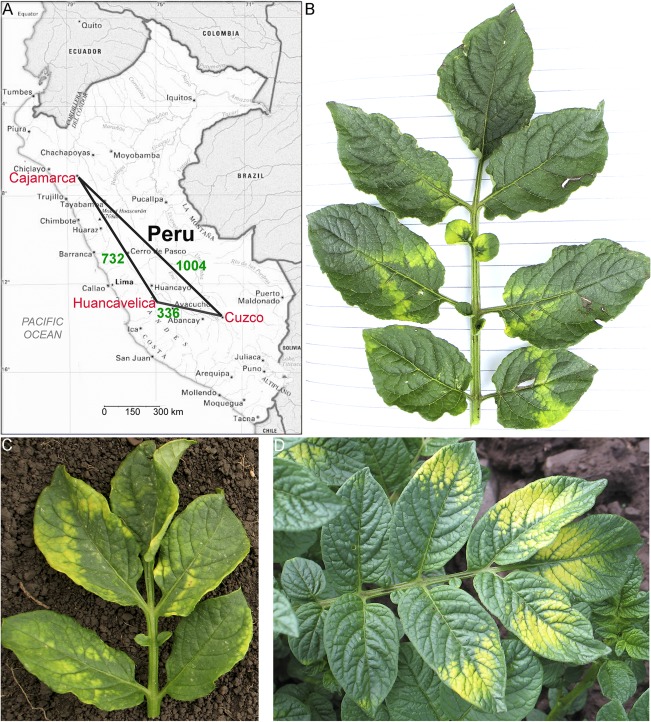
Geographical locations of *Potato mop‐top virus* (PMTV) isolates collected in Peru and the appearance of symptoms on potato leaves of the sampled plants. (A) Map shows three geographical locations in which samples of potato leaves were collected. Numbers on the edges of the scalene triangle indicate the distance in kilometres between Cuzco, Huancavelica and Cajamarca. (B, C) Leaves of potato plants displaying yellow chevron‐like symptoms typical of PMTV infection. (D) Leaves of potato plant displaying bright yellow symptoms.

To determine the genetic composition of PMTV isolates in Peru (Fig. 1A), leaves of Andean potato plants that showed yellow ‘chevron’‐like symptoms (yellow V‐shaped patterns; Fig. 1B–D) were collected during the rainy season (February–March 2009 and 2011; Table S1, see Supporting Information) in three locations (Cuzco, Huancavelica and Cajamarca areas; Fig. 1A and Table S1). Two or three leaf samples with similar symptoms from the same field were pooled. Total RNA was extracted from 41 pools of plants and subjected to reverse transcription‐polymerase chain reaction (RT‐PCR) with PMTV‐specific primers to amplify full‐length RNA‐TGB. In parallel experiments, the same samples were analysed by enzyme‐linked immunosorbent assay (ELISA) using monoclonal antisera against PMTV CP. Twelve samples appeared to be positive for the presence of RNA‐TGB, indicating that the plants were infected with PMTV. The RT‐PCR data correlated with the ELISA data; however, only six plants were positive for PMTV CP antigen, showing that RT‐PCR is a more analytically sensitive method than ELISA. The symptoms shown in Fig. 1B,C always correlated with the presence of PMTV in the leaves (seven of seven plants). In contrast, not all plants showing bright yellow symptoms (Fig. 1D) were positive for PMTV infection (five positive of 34 analysed).

**Figure 2:**
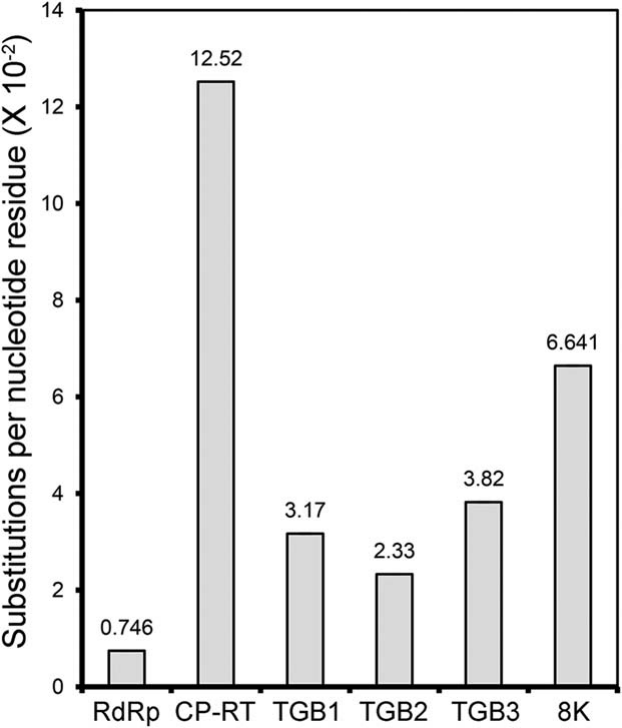
Number of substitutions per site identified in each genomic region determined according to single‐likelihood ancestor counting (SLAC) analysis of the sequences of Peruvian isolates of *Potato mop‐top virus* (PMTV).

Three full‐length PMTV genomic components were amplified using primers specific for well‐conserved 5′‐ and 3′‐termini. Notably, the sequences of the forward primers contained the sequence of the bacteriophage T7 promoter to facilitate biological characterization of the isolates following cloning, *in vitro* transcription and plant inoculation with the transcripts. Between nine and 30 clones for each full‐length genomic RNA component were sequenced (Table S2, see Supporting Information). The genetic variability of three genomic components together accounted for a total of 716 mutations (base substitutions, nonsense mutations and indels) identified by a single nucleotide polymorphism (SNP) analysis algorithm implemented in the DNASTAR Lasergene Core Suite in 61 clones (Table S2), corresponding to 213 105 nucleotides sequenced. The *CP‐RT* and *8K* cistrons accumulated the largest number of mutations (12.52 × 10^−2^ and 6.641 × 10^−2^ substitutions per nucleotide residue position, respectively; Fig. 2), whereas the replicase region harboured the lowest number of mutations (Fig. 2). Altogether, these results indicate that the accumulation of mutations was not evenly distributed among the six genomic regions of PMTV that were analysed.

### Direction of selective forces on the PMTV genome

To address the question of whether positive or negative selection is shaping PMTV populations, synonymous mutations per synonymous site (*d*_S_), non‐synonymous mutations per non‐synonymous site (*d*_N_) and the *d*_N_/*d*_S_ ratio were calculated by single‐likelihood ancestor counting (SLAC) analysis using Datamonkey (Kosakovsky Pond and Frost, 2005a, 2005b). As shown in Table 1, the *d*_N_/*d*_S_ ratio for each cistron, estimated individually, ranged from 0.082 (*TGB2*) to 1.415 (*8K*), suggesting different constraints on sequence change depending on the virus gene. Accordingly, the RdRp, CP‐RT and TGB genomic regions contained substitution spectra subjected to negative selection (*d*_N_/*d*_S_ < 1), consistent with the fact that the genes are essential for infectivity and the encoded proteins are involved in virus replication, encapsidation/transmission and movement, respectively (Reavy *et al*., 1998; Zamyatnin *et al*., 2004).

**Table 1:** Estimated *d*_N_/*d*_S_ ratio for different cistrons of the *Potato mop‐top virus* (PMTV) genome.

	*d*_N_/*d*_S_ ratio	
Gene	Peruvian isolates	All available isolates
*RdRp*	0.141	0.145
*CP*	0.123	0.261
*CP‐RT*	0.414	0.415
*TGB1*	0.173	0.187
*TGB2*	0.061	0.082
*TGB3*	0.216	0.191
*8K*	1.863	1.415

Notably, the *d*_N_/*d*_S_ ratios for *TGB1* (*d*_N_/*d*_S_ = 0.187) and *TGB3* (*d*_N_ */d*_S_ = 0.191) were very similar, suggesting similar stringent constraints on amino acid (AA) residue change as the effect of purifying selection. However, the *TGB2* cistron, which significantly overlaps with *TGB1* and *TGB3*, had the lowest *d*_N_/*d*_S_ ratio (*d*_N_/*d*_S_ = 0.082), indicating even further constraints on AA residue change.

In contrast, *8K* was found to be subjected to strong positive selection (*d*_N_/*d*_S_ = 1.415; *d*_N_/*d*_S_ > 1), consistent with the fact that *8K* is dispensable for virus infectivity and long‐distance movement (Savenkov *et al*., 2003), but is needed for efficient virus accumulation (Lukhovitskaya *et al*., 2013). Taken together, these results indicate that PMTV RdRp, CP‐RT and TGB genomic regions are under negative selection, whereas the *8K* cistron is under diversifying (positive) selection.

### Phylogenetic analysis of Peruvian PMTV isolates relative to those characterized previously

**Figure 3:**
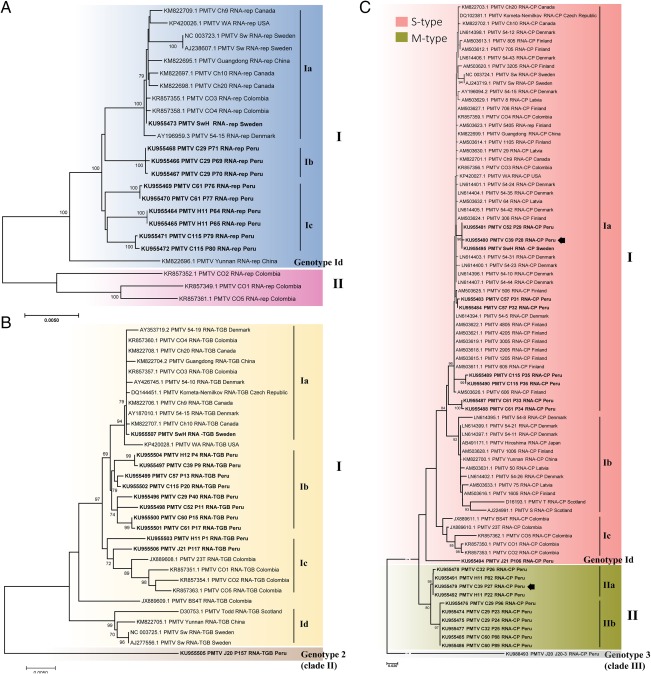
Phylogenetic relationships among sequences of RNA‐rep (A), RNA‐TGB (B) and RNA‐CP (C) genomic components of *Potato mop‐top virus* (PMTV) inferred by the neighbour‐joining (NJ) method from multiple sequence alignments. (A) The NJ tree of 23 aligned complete RNA‐rep sequences shows two clades. (B) The NJ tree of 31 aligned complete RNA‐TGB sequences shows one clade and one novel genotype. (C) The NJ tree of 79 aligned RNA‐CP sequences shows two clades and one novel genotype. The percentages (greater than 70%) of replicate trees in which the associated sequences clustered together in the bootstrap test (1000 replicates) are shown next to the branches. The arrow indicates the recombinant RNA‐CP sequence found in clones P33 and P34 (field code/isolate C61).

To determine the phylogenetic relationships of 61 full‐length Peruvian PMTV sequences in relation to the known PMTV genomes from GenBank, we performed multiple sequence alignments and phylogenetic analysis with two statistical methods: maximum likelihood (ML) and neighbour‐joining (NJ) methods. Both ML and NJ approaches yielded virtually identical results (data not shown). Identical sequences derived from a single isolate were excluded. Figure 3 illustrates the NJ bootstrap consensus trees representing phylogeny within the three genome components. The inferred tree for RNA‐CP showed the maximum bifurcation, whereas the RNA‐rep tree exhibited the lowest branching of all PMTV genomic components (Fig. 3).

The RNA‐rep bootstrap consensus tree revealed two major clades which have been described previously (Gil *et al*., 2016). The Peruvian isolates grouped together with isolates from Colombia, Europe, Canada and USA in clade I, but into distinct subclades Ib and Ic (Fig. 3A). As reported previously (Gil *et al*., 2016), clade II was exclusively represented by Colombian isolates. The Peruvian isolates shared 97% identity with clade II.

Phylogenetic analysis of RNA‐TGB identified one major clade, clade I, and one novel distinct branch, which was represented by a single isolate, J20‐P157, from Cajamarca and provisionally termed genotype 2. Ten other Peruvian isolates fell into clade I, eight of which grouped together into a subclade distinct from the sequences from Europe, North America, China and Colombia, whereas two isolates, namely H11‐P1 and J21‐P117, grouped along with isolates from Colombia in subclade Ic (Fig. 3B). Genotype 2 (isolate J20‐P157) shared 92%–94% identity with the isolates of clade I.

Phylogenetic analysis of RNA‐CP grouped the isolates into two clades and one novel distinct branch, which was represented by an isolate from Cajamarca and provisionally termed genotype 3 (Fig. 3C). Genotype 3 shared 80% identity with isolates within clade I and clade II. Peruvian isolates grouped into three lineages (two clades and one genotype), indicating higher variability of RNA‐CP in Peru than in other parts of the world. Furthermore, inclusion of Peruvian isolates into the phylogenetic analysis showed that clades I, II and III described previously (Gil *et al*., 2016) clustered together in the newly identified clade I, and therefore were provisionally termed strain S (or S‐type), whereas clade II was provisionally termed strain M (or M‐type). The reasons for this distinction are explained below.

### Analysis of the 8K protein sequences

**Figure 4:**
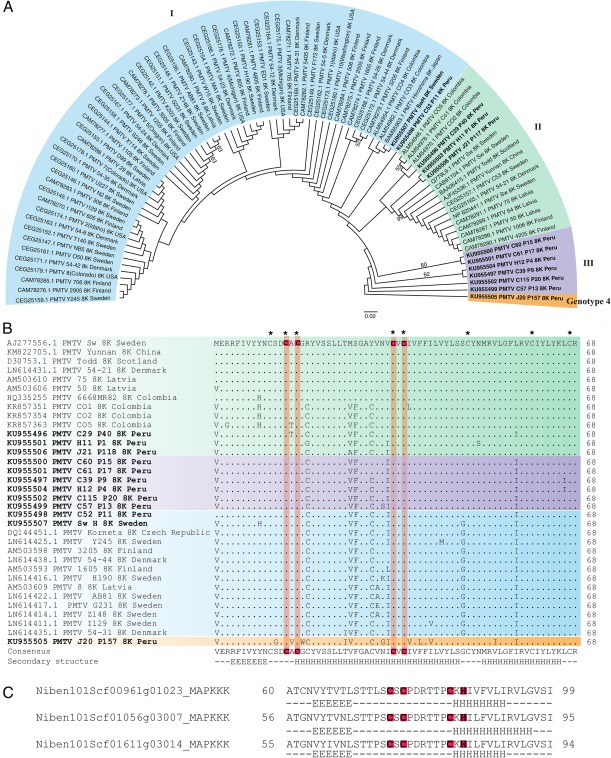
Analysis of structural motifs, variability and phylogenetic relationships among sequences of the 8K protein. (A) The neighbor‐joining (NJ) tree of amino acid sequences of the 8K protein from 84 isolates shows three clades and one novel genotype. The evolutionary distances were computed using the Poisson correction method. (B) Multiple alignment showing amino acid differences in the sequence of the 8K protein. Identical sequences of 8K belonging to the isolates from the same country are not shown in the figure. Conserved cysteine residues are indicated with an asterisk, whereas those involved in the formation of the putative SWIM zinc‐finger motif C*x*C*x_n_*C*x*C are highlighted. Secondary structure prediction is shown below the sequence. (C) Secondary structure prediction of three SWIM motif‐containing mitogen‐activated protein kinases (MAPKKK) from *Nicotiana benthamiana*. (B, C) Protein secondary structures were predicted using the JPred4 program. H, α‐helix; E, β‐strand.

As the 8K genomic region exhibits high variation and is under positive selection, as established in this study, we performed multiple sequence alignment and phylogenetic analysis of the 8K protein sequences (Fig. 4A,B). The 8K bootstrap consensus tree showed three clades, and one novel distinct branch, which was represented by an isolate from Cajamarca and provisionally termed genotype 4 (Fig. 4A). Most of the 8K sequences from Latvia, Sweden, Denmark, Finland, USA and Japan clustered together with two Colombian and one Peruvian isolate in clade I, whereas three Peruvian 8K sequences fell into clade II, together with sequences from Colombia, the Nordic countries, Latvia and China. Clade III was exclusively represented by the 8K sequences from Peru. Clade I shared 89%–98% identity with clade II, 88%–95% identity with clade III and 77%–85% identity with genotype 4. Notably, the 8K sequence of the isolate from Cajamarca (genotype 4) shared as little as 77% identity with the other isolates, the lowest identity ever reported for pomoviruses of the same species.

Previously, 8K has been described as a ‘cysteine‐rich’ protein because the protein is enriched for cysteine residues (Lukhovitskaya *et al*., 2005). However, previous attempts to predict zinc‐finger motifs within 8K have failed because of the large number of cysteine residues (eight to ten depending on the isolate) within the sequence and their poor conservation among limited numbers of isolates. With the availability of 86 8K sequences from various geographical locations, it is possible to identify the conserved cysteine residues, which appear to be C_11_, C_14_, C_16_, C_34_, C_36_, C_48_, C_60_ and C_67_, whereas C_18_ and C_30_ are not conserved in all of the isolates analysed (Fig. 4B). Among these conserved residues, only C_14_ and C_16_ on the one hand, and C_34_ and C_36_ on the other, are separated by an equal number of other AA residues, one AA in this case. Notably, this type of residue distribution pattern is typical of SWIM zinc‐finger motifs with a consensus sequence C*x*C*x_n_*C*x*H (where *x* denotes any AA; Makarova *et al*., 2002). The predicted secondary structure using the JPred 4 server (Drozdetskiy *et al*., 2015) further supported the presence of the SWIM motif, although the putative SWIM motif of the 8K protein deviated slightly from the classical sequence (C*x*C*x_n_*C*x* **H**), with a cysteine residue replacing histidine (C*x*C*x_n_*C*x* **C**). Analysis of *Nicotiana benthamiana* genome sequences identified the presence of the SWIM motif in three mitogen‐activated protein kinases (MAPKKK; Fig. 4C).

### Further analysis of CP‐RT sequences: evidence for recombination and identification of internal in‐frame deletions

**Figure 5:**
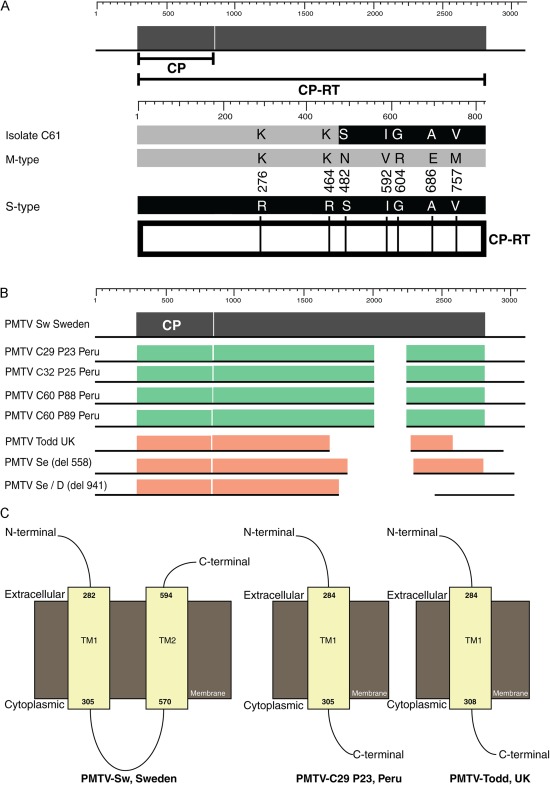
Analysis of *Potato mop‐top virus* (PMTV) CP‐RT sequences. (A) Schematic representation of the CP‐RT proteins encoded by M‐type (grey‐shaded box) and S‐type (black‐shaded box) RNA‐CP segments, as well as by the putatively recombined RNA‐CP segment of isolate C61. Seven signature amino acid residues of M and S types and their positions are shown. (B) Schematic representation of internal in‐frame deletions in the CP‐RT region of some Peruvian isolates relative to CP‐RT of the Swedish isolate (Sw) and Scottish isolate Todd (T). (C) Predicted topology of the CP‐RT proteins in the cell membrane. Transmembrane helices are depicted as yellow boxes. The transmembrane domains (TM) were predicted using the Phyre2 server.

As 10 Peruvian isolates grouped together in a distinct clade II (M‐type), which differed from the rest of the PMTV RNA‐CP sequences worldwide, all available CP‐RT protein sequences were aligned to identify possible signature AAs for clade I (S‐type) and clade II (M‐type). We found seven AA positions, which were conserved in all clade I (S‐type) isolates, but differed from the corresponding residues of the clade II (M‐type) isolates (Fig. 5A). These molecular signatures were located within the read‐through domain of CP‐RT (Fig. 5A). This analysis also revealed that one of the isolates, namely C61‐P33 (and also clone C61‐P34), seemed to be a recombinant between M and S types (Fig. 5A). For further characterization, recombination detection analyses were performed using all available sequences for PMTV RNA‐rep, RNA‐CP and RNA‐TGB. The recombination detection program did not propose recombination events (data not shown). For recombination to occur, two different strains must replicate in the same cell. To establish whether this could be the case for PMTV, further inspection of the phylogenetic tree was performed. We found that the sequences of two clones, C39‐P27 and C39‐P28, obtained from the same field (field code C39) differed significantly (97% identity), with C39‐P27 grouping together with M‐type isolates, whereas C39‐P28 clustered together with S‐type isolates. This observation suggests that recombination between S‐ and M‐type RNA‐CP could potentially occur, as S‐ and M‐type RNA‐CP sequences were found in the same field.

Multiple sequence alignment of available sequences for the CP‐RT protein also revealed that three Peruvian isolates, C29‐P23/P24, C32‐P25 and C60‐P88 (and clone C60‐P89), contained internal in‐frame deletions (Fig. 5B) spanning the region in *CP‐RT*, which was previously reported as being deleted in PMTV laboratory isolate Todd, as well as in Swedish isolates propagated by serial passages or identified in the field (Sandgren *et al*., 2001). However, the Todd isolate had a longer deletion relative to Peruvian isolates (Fig. 5B). The presence of a 76 AA deletion in C29‐P23/P24, C32‐P25 and C60‐P88/89 prompted us to further analyse CP‐RT. Using Phyre2 and TMHHM algorithms, two transmembrane domains were predicted, TM1 and TM2, spanning regions AA 282–305 and AA 570–594, respectively (Fig. 5C). The CP‐RT of the most divergent isolate from Cajamarca was predicted to contain similar transmembrane segments spanning AA 283–305 and AA 571–595 (Fig. S1, see Supporting Information). These data suggest that CP‐RT is a membrane protein inserted into the lipid bilayer in a U‐shaped orientation (Fig. 5C). Following hydrophobicity analysis of the C32‐P23/P24, C32‐P25 and C60‐P88/89 isolates, the TM2 domain appeared to be lacking in the CP‐RT protein owing to internal in‐frame deletions in the CP‐RT open reading frame (ORF) (Fig. 5C).

### Pathobiological properties of Peruvian isolates

The phylogenetic analysis described above helped us to define at least two distinct clades based on the RNA‐CP sequence, namely M‐ and S‐type. However, it remained unclear whether there were biological differences between isolates containing M‐ and S‐type RNA‐CP segments. Therefore, we sought to determine whether various pathobiological properties of isolates were associated with the presence of either M‐ or S‐type RNA‐CP segments.

**Figure 6:**
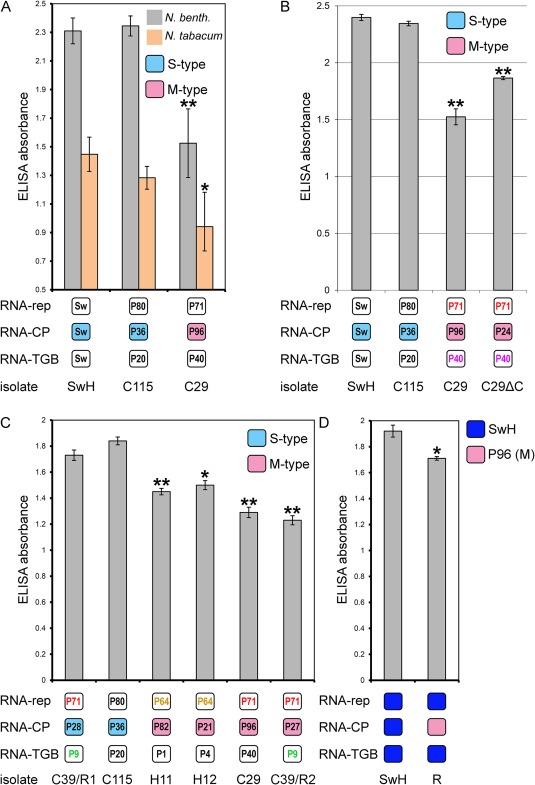
Differences in *Potato mop‐top virus* (PMTV) accumulation in the leaves of *Nicotiana benthamiana* and *Nicotiana tabacum* cv. Samsun plants systemically infected with PMTV isolates and reassortants possessing either an S‐ or M‐type RNA‐CP segment. The data represent means (bars) ± standard deviation (A) or means (bars) ± standard errors of the mean (B–D) of the results determined with six samples for each isolate derived from two independent experiments performed in triplicate. *P < 0.01; **P < 0.005; Student's *t*‐test. Blue boxes represent S‐type RNA‐CP segments, whereas pink boxes represent M‐type RNA‐CP segments. The numbers of the clones used to generate *in vitro* transcripts are shown inside the boxes; the same clones are shown by the same colour. Field codes (isolates) are shown below the boxes. R, reassortant.

We took advantage of the availability of the complete cDNA clones for all new isolates characterized above. In addition, infectious cDNA clones were available for a Swedish isolate of the virus (Savenkov *et al*., 2003), which appeared to have an S‐type RNA‐CP segment (Fig. 3). In these and subsequent experiments, RNA‐CP transcripts from M‐ and S‐type RNA‐CP cDNA clones were prepared and inoculated onto *N. benthamiana* and *N. tabacum* leaves, together with transcripts of RNA‐rep and RNA‐TGB, and the type of inoculum used in each case was referred to by the field code. At 14 days post‐inoculation (dpi), no symptoms were observed on *N. tabacum* plants inoculated with the Swedish isolate (S‐type) or Peruvian isolate C115 (S‐type) or Peruvian isolate C29 (M‐type; data not shown), consistent with the previous reports showing that PMTV causes symptomless systemic infection in *N. tabacum* (Torrance *et al*., 2009). In contrast, in *N. benthamiana*, Swedish and Peruvian isolate C115 induced yellow mosaic symptoms typical of PMTV infection, whereas Peruvian isolate C29 (M‐type) caused much milder symptoms (Fig. S2, see Supporting Information). To further assess whether the differences in pathogenesis were caused by a difference in the CP‐RT sequence, we quantified virus accumulation in the upper systemically infected leaves of *N. benthamiana* and *N. tabacum* plants at 14 dpi. Significant differences in virus accumulation were observed (Fig. 6A) as ELISA values for Peruvian isolate C29 (M type) were, on average, 44% (*P* < 0.005) lower in *N. benthamiana* and 46% (*P* < 0.01) lower in *N. tabacum* relative to the ELISA values for the Swedish and C115 isolates (both S‐type; Fig. 6A). The PMTV ELISA values in the systemically infected leaves were similar (*P* > 0.05) between the Swedish and Peruvian isolate C115. As the differences in virus accumulation between S‐ and M‐types were similar in *N. benthamiana* and *N. tabacum*—44% versus 46%, respectively—subsequent experiments were conducted in *N. benthamiana*.

To address the question of whether an internal in‐frame deletion in the CP‐RT region affects virus accumulation, two different inocula were assembled comprising RNA‐rep + RNA‐TGB + RNA‐CP (P96 clone of RNA‐CP; collectively referred to as C29) and RNA‐rep + RNA‐TGB + RNA‐CP (P24 clone of RNA‐CP carrying a deletion; referred to as C29ΔC). Additional inocula were assembled for the Swedish isolate and the Peruvian isolate C115 to serve as controls. Quantitative analysis of progeny virus accumulation at 14 dpi revealed that the ELISA absorbance values of the deletion variant C29Δ progeny were approximately 23% (*P* < 0.005) higher in the upper leaves than were those of C29 (Fig. 6B). Nevertheless, as before, the ELISA absorbance values of the Swedish isolate and Peruvian isolate C115 (both S‐type) were approximately 62%–87% higher than those of C29 and C29Δ (both M‐type; Fig. 6B).

As a next step, we wanted to further confirm that the RNA‐CP genome segment was responsible for the differences in virus accumulation. To this end, two S‐type RNA‐CP‐containing inocula and four M‐type RNA‐CP‐containing inocula were assembled (Fig. 6C), inoculated on the lower leaves of *N. benthamiana* plants and the upper leaves of the plants were analysed by ELISA at 14 dpi. As before, the RNA‐CP segment had the largest impact on pathogenesis. PMTV isolates containing the M‐type RNA‐CP segment accumulated at significantly lower levels (H11, H12, C29, C39/R2: *P* < 0.005; H12: *P* < 0.01) relative to PMTV isolates containing the S‐type RNA‐CP segment (Fig. 6C). Moreover, substitution of the S‐type with the M‐type RNA‐CP segment also reduced (*P* < 0.001) the virus titre (Fig. 6C, compare single reassortants C39/R1 and C39/R2; Fig. 6D). Altogether, the data show that CP‐RT is the major determinant of increased/decreased viral load and severity of the virus‐induced symptoms/disease.

## Discussion

PMTV continues to pose a significant threat to potato production, as exemplified by the recent recognition of the presence of the virus in Canada (Hu *et al*., 2016) and the USA (Ramesh *et al*., 2014). Therefore, there is a growing need to understand the variability of the virus at a global scale. Previous studies examining the genetic composition of PMTV isolates in Europe, Asia and North America have reported low variability of the virus (Beuch *et al*., 2015; Hu *et al*., 2016; Ramesh *et al*., 2014). However, it remains unclear whether low variability of PMTV is typical for all parts of the world. Therefore, we sought to determine PMTV diversity in the Andean region of South America, the centre of potato domestication. Our study shows great variability of the virus in the Andean regions of Peru.

In this study, 12 Peruvian field isolates of PMTV sampled in 2009 and 2011 were characterized by nucleotide sequencing of 61 full‐length cDNA clones, amounting to a total of 213 105 nucleotides. Genetic variation of naturally occurring isolates can be overestimated because of experimental errors introduced through the process of copying and amplification of viral RNA. The errors might arise from the use of low‐fidelity polymerases (Domingo *et al*., 2006). To avoid such bias, the following precautions were taken: (i) a Phusion high‐fidelity DNA polymerase was used for PCR amplification; (ii) full‐length genome segments were amplified using short primers specific for 5′‐ and 3′‐ends to avoid the subsequent need for the assembly of full‐length PMTV sequences from partial clones; and (iii) the reaction conditions were optimized to favour the amplification of most of the virus variants. Indeed, the utility of such an approach was justified by the following observations. When two to five clones for each isolate were subjected to sequencing, the sequences of the parallel clones of the same isolate appeared to be identical, whereas the sequences differed between the isolates (Table S2; roman type versus italic type). However, we failed to amplify full‐length cDNAs for the RNA‐rep segment, the longest PMTV genome component, from eight isolates, because of problems with logistics as many potato leaf samples sent by local courier arrived in Lima in extremely poor condition.

Our results showed the uneven distribution of nucleotide base differences in the six genomic regions analysed, with the highest mutation rates found in the *CP‐RT* and *8K* cistrons. Selection pressure acting on *RdRp*, *CP‐RT*, *TGB1*, *TGB2*, *TGB3* and *8K* genes was assessed by measuring the average *d*_N_/*d*_S_ values, indicating that *RdRp*, *CP‐RT*, *TGB1*, *TGB2* and *TGB3* were under purifying selection (*d*_N_/*d*_S_ < 1). However, the *d*_N_/*d*_S_ value of the *8K* gene was found to be 1.415 (*d*_N_/*d*_S_ > 1), which identified the *8K* gene as being under strong positive (diversifying) selection, suggesting acceleration in the divergence of the *8K* gene in increasing the adaptability of the virus. It is noteworthy that the previous analysis of the *8K* gene from European isolates and some isolates from Colombia did not find that positive selection shapes the evolution of the *8K* sequence, as the reported *d*_N_/*d*_S_ value was 1.100 (Beuch *et* al., 2015). In contrast, when *8K* from Peruvian isolates only was subjected to SLAC analysis, the *d*_N_/*d*_S_ value was found to be even higher, 1.863. The phylogenetic analysis showed that variability of the *8K* sequence among Peruvian isolates is much greater than the sequence variability in Europe, Asia and North America. The reason for this is not known, but could imply adaptation of the virus to the native potato varieties in Peru and/or different potato species and/or an accelerated rate of PMTV evolution owing to the great genetic variation of potato and its wild relatives in this region. Indeed, according to the CIP website, farmers in Peru grow more than 4000 edible potato varieties/species. The importance of the great variability of *8K* is currently unknown, but this genetic plasticity or structural flexibility of the encoded protein should be evaluated in future studies.

Phylogenetic analysis indicated that the PMTV isolates sequenced so far were divided into two to three lineages depending on the genomic segment. In all lineages, except one (RNA‐rep clade II), there were virus isolates from Peru demonstrating greater variability of the virus in Peru relative to other parts of the world. Notably, clade II of RNA‐rep was exclusively represented by isolates from Colombia. This was similar to the previous study, which classified PMTV RNA‐rep into two clades (Gil *et al*., 2016). The inclusion of the 61 sequences of Peruvian isolates helped to more clearly define the lineages for each genomic segment, namely the RNA‐TGB segment was classified into two clades, whereas RNA‐CP was classified into three clades. This differs from previous studies (Gil *et al*., 2016; Santala *et al*., 2010), which singled out three clades for each of the RNA‐TGB and RNA‐CP segments, with identity as high as 97%–98% between the clades. In the current grouping, which was supported by the bootstrap tests, the clades share 80%–95% identity with each other.

In the current study, we suggest a novel classification of PMTV isolates based on genotype constellations (Table 2). In this classification, the genotype clade of each RNA segment is taken into consideration. Accordingly, the isolates for which complete genome sequences are available can be catalogued into four genetic constellations (Table 2). Notably, three of the four genetic constellations were found in Peru, and another unique genetic constellation was identified in Colombia. However, isolates from Colombia, USA, Canada, China, Denmark, Sweden and some Peruvian isolates were found to belong to a single genetic constellation (I + I + I), suggesting that this particular genetic constellation was initially introduced to Europe and/or subsequently spread to other parts of the world. This notion is supported by a study of the global genetic diversity of *S. subterranea*, the soil‐borne vector of PMTV (Gau *et al*., 2013). Estimations of gene flow based on microsatellite and DNA sequence data suggest that *S. subterranea* was probably introduced to Europe from South America and, subsequently, Europe served as a source of migrants of the pathogen for world‐wide dissemination (Gau *et al*., 2013). Moreover, South American populations of *S. subterranea* (132 samples from four countries analysed) were found to have consistently greater diversity than those from other parts of the world (Gau *et al*., 2013). Considering that PMTV dissemination depends on its vector, these data are in line with the high genetic diversity of Peruvian isolates of PMTV reported in this article.

**Table 2:** *Potato mop‐top virus* (PMTV) genetic constellations inferred for reported sequences (including this study).

Genetic constellation	PMTV isolates
I* + I^†^ + I^‡^	Peru C115; Colombia CO3, CO4; USA WA; Canada Ch9, Ch10, Ch20; China Guangdong, Yunnan; Denmark 54‐15; Sweden Sw, SwH
I + I + II	Peru H11, C29, C61
II + I + I	Colombia CO1, CO2, CO5
?^§^ + II + III	Peru J20

*RNA‐rep genotype clade according to phylogenetic analysis.

^†^RNA‐TGB genotype clade.

^‡^RNA‐CP genotype clade.

^§^Sequence for RNA‐rep is not available.

We sought to determine the potential for pathobiological differences between isolates. The phylogenetic analysis indicated that sequences of the RNA‐CP segment grouped on the basis of their geographical origin and showed greater variability relative to the sequence variation of RNA‐rep and RNA‐TGB segments. Our studies, using plant inoculation with infectious clones, identified a clear bias towards reassortment of the RNA‐CP genome segment. We found that single reassortant viruses containing M‐type RNA‐CP segments accumulated at lower levels than viruses containing S‐type segments. Thus, exchange of the RNA‐CP segment from M‐type to S‐type increased the virus titre. These single‐segment reassortants accumulated at levels similar to the four isolates with an M‐type RNA‐CP segment. However, the three PMTV isolates containing an S‐type RNA‐CP segment accumulated at higher levels relative to the titres of the mild isolates (M‐type). Characterization of single‐segment reassortants also demonstrated that reassortant viruses moved systemically in the infected plants, suggesting that the newly formed genomic constellations are genetically and functionally stable.

Phylogenetic analysis and molecular characterization of reassortant viruses suggested that the read‐through domain of CP‐RT was a major determinant of the pathobiological properties of different isolates, because all AA differences between S‐ and M‐types were located in this region. In this study, we identified seven signature AA differences in the read‐through region of CP‐RT encoded by M‐ and S‐types. It remains to be determined whether and which of these AA residue substitutions are associated with differences in the accumulation of severe and mild isolates. At this time, we cannot address whether the observed differences in virus accumulation are caused by differences in the rate of virus systemic movement, CP‐RT protein localization or altered protein half‐life/stability/turnover. These questions will be addressed in further studies.

Our results highlight the potential for environmental reassortment and/or mixed infections with M‐ and S‐type genome segments that can lead to recombination between M‐ and S‐types. Indeed, as reported in this study, both M‐ and S‐type segments were cloned from one of the field samples, suggesting that mixed infection with at least two RNA‐CP genotypes could be possible. Moreover, an RNA‐CP segment cloned from total RNA extracted from a sample from another field appeared to be a recombinant between M‐ and S‐types. These findings support the potential for the emergence of novel genotypes of the virus in the Andean regions of South America when compared with the rest of the world, from which only S‐type RNA‐CP has been reported so far.

We observed the presence of deleted forms of RNA‐CP in samples collected from three fields. Although both full‐length and deleted forms were cloned from two fields (field codes C29 and C32), we were unable to detect the presence of untruncated RNA‐CP in one of the fields (field code C‐60; data not shown). This might indicate that the deletions occurred at the very early stages of infection before sampling was conducted or, alternatively that deletion may have already existed in the infected potato plants and the deletion variants were transmitted by vegetative propagation through infected tubers. The second scenario seems to be possible as deletions in the CP‐RT protein, which is encoded by RNA‐CP, have been shown to prevent transmission of PMTV by its natural vector *S. subterranea* (Reavy *et al*., 1998). Previous studies have demonstrated that CP‐RT deletions often occur when PMTV is transmitted mechanically by multiple passages from plant to plant (Sandgren *et al*., 2001). In this respect, serial mechanical passages of the virus seem to be analogous to vegetative propagation of the virus through tubers, and result in similar types of deletions, which remove one (TM2) of two transmembrane segments predicted for the CP‐RT protein. Notably, CP‐RT deletion found in C29‐P24 versus C29‐P96 was associated with a slight increase in virus accumulation, suggesting that shorter RNA‐CP species replicate faster than the wild‐type. Previous studies have also demonstrated that deletions in the central part of CP‐RT, resulting in the loss of TM2 segments, do not affect virus systemic movement (Torrance *et al*., 2009). It remains to be determined whether TM1 and TM2 are needed for transmission of the virus by its natural vector *S. subterranea*.

Taken together, we have established that, in Peru, PMTV has undergone continual evolutionary divergence, resulting in the establishment of distinct phylogenetic clades, genotypes and genetic constellations. Our results highlight the potential for environmental reassortment of PMTV genomic segments in South America, which can lead to novel genotype constellations with potential epidemiological risks. Further studies of these isolates and other single‐reassortant viruses are required to understand the constellation of reassortants, which could represent epidemiological risks, and to understand the degree of functional compatibility/superinfection exclusion (Folimonova *et al*., 2014) between segments.

## Experimental Procedures

### Virus isolates

Leaves of potato plants showing yellow symptoms (Fig. 1B–D) were collected in potato fields in February–March 2009 and 2011 in Cuzco, Huancavelica and Cajamarca areas in Peru (Table S1), wrapped into plastic bags and sent by a local courier to CIP, Lima, Peru. On arrival, the samples were stored at −70 °C. Leaves from two or three plants were pooled into the same plastic bag.

### ELISA

Double‐antibody sandwich (DAS)‐ELISA to detect and quantify PMTV was performed on equal amounts of leaf samples using anti‐CP monoclonal antibodies and anti‐CP monoclonal alkaline phosphatase conjugate, according to the manufacturer's instructions ( BioReba AG, 4153, Reinach, Switzerland). Absorbance was measured at 405 nm.

### RT‐PCR, cloning and sequencing

Total RNA was extracted from pooled leaf samples using an RNeasy Plant Mini kit (Qiagen GmbH, 40724, Hilden, Germany (Line 653‐Qiagen AB.) according to the manufacturer's instructions. One microgram of total RNA was used for cDNA synthesis with Superscript III reverse transcriptase (Thermo Fisher Scientific, 02241, Vilnius, Lithuania,USA). An aliquot of cDNA was subsequently used as a template for PCR. The PCRs were performed using Phusion Flash High‐Fidelity PCR Master mix (Thermo Fisher Scientific, 02241, Vilnius, Lithuania,USA) with PMTV‐specific primers (Table S3, see Supporting Information). In order to generate full‐length infectious clones, the cDNAs were amplified with forward primers containing the sequence of the T7 promoter. PCR was performed with an initial denaturing step for 1 min at 98 °C, followed by 35 cycles consisting of 98 °C for 20 s, 54 °C for 20 s, 72 °C for 1 min 30 s, and final elongation at 72 °C for 7 min. PCR products were incubated with Taq polymerase in the presence of dT to facilitate TA‐cloning. The purified PCR fragments were ligated into a pGEM‐T Easy Vector System (Promega corporation, Madison, WI‐53711‐5399, USA). The obtained full‐length infectious cDNA clones were sent for sequencing to Macrogen Europe, Amsterdam, The Netherlands.

### Sequence analysis

Sequences were analysed with DNASTAR Lasergene Core Suite, version 12 (DNASTAR, Inc., Madison, WI, USA). DNA sequences were aligned by ClustalW using the IUB DNA weight matrix, whereas protein sequences were aligned by MUSCLE implemented in MEGA 7. Phylogenetic analyses were conducted using the NJ method implemented in MEGA 7 (Kumar *et al*., 2016). Genetic distances were estimated using the Tamura–Nei model in MEGA 7 and standard deviations were calculated by bootstrapping with 1000 replicates.

Synonymous substitutions per synonymous site (*d*_S_) and non‐synonymous substitutions per non‐synonymous site (*d*_N_) were calculated using SLAC analysis implemented in Datamonkey (http://www.datamonkey.org/; Kosakovsky Pond and Frost, 2005a).

Recombination analysis was carried out for the whole genome as well as individual ORFs using Recombination Detection Program, RDP v 4.56 (Martin *et al*., 2015).

The secondary structures of the 8K and MAKKK proteins were predicted using the Jpred 4 server (http://www.compbio.dundee.ac.uk/jpred4/; Drozdetskiy *et al*., 2015). Transmembrane domains were predicted using the Phyre2 web portal (http://www.sbg.bio.ic.ac.uk/~phyre2; Kelley *et al*., 2015) and the TMHMM prediction algorithm (http://www.cbs.dtu.dk/services/TMHMM/; Krogh *et al*., 2001).

### Plant growth conditions

*Nicotiana benthamiana* and *N. tabacum* plants were grown in a Phytotron growth facility with a day length of 16 h, daytime temperature of 20 °C and night‐time temperature of 18 °C.

### Plant inoculation with *in vitro* transcripts

*In vitro* transcripts were synthesized on *Mlu*I‐linearized RNA‐rep and RNA‐CP clones (plasmids), and *Spe*I‐linearized RNA‐TGB plasmids. *In vitro* transcription was performed using a T7‐RiboMAX large‐scale RNA production system (Promega) in the presence of m7GpppG cap analogue (New England Biolabs, Ispwich, MA‐01938‐2733, USA) according to the manufacturer's instructions. Transcripts were then mixed with GKP buffer [50 mm glycine, 30 mm K_2_HPO_4_ (pH 9.4), 1% (w/v) bentonite and 1% (w/v) celite] in a 1 : 1 : 1 : 1 ratio and rub inoculated onto *N. benthamiana* leaves.

## Supporting information

Additional Supporting Information may be found in the online version of this article at the publisher's website:

**Fig. S1** Predicted transmembrane domains in the CP‐RT region of the J20 isolate using the Phyre2 server.

**Fig. S2** Symptoms induced in *Nicotiana benthamiana* by *Potato mop‐top virus* (PMTV) isolates carrying either severe (S) or mild (M) type of RNA‐CP segment.

**Table S1** Geographical locations and GenBank accession numbers of the Peruvian isolates characterized in this study.

**Table S2** Full‐length cDNA clones (plasmids) of Peruvian isolates of *Potato mop‐top virus* (PMTV).

**Table S3** List of primers used in this study.
