# Supplementary figures and images for: Molecular and pathobiological characterization of 61 Potato mop‐top virus full‐length cDNAs reveals great variability of the virus in the centre of potato domestication, novel genotypes and evidence for recombination
Source: Mol Plant Pathol. 2017 May 11;18(6):864–77. doi: 10.1111/mpp.12552 (PMC6638219; doi:10.1111/mpp.12552)

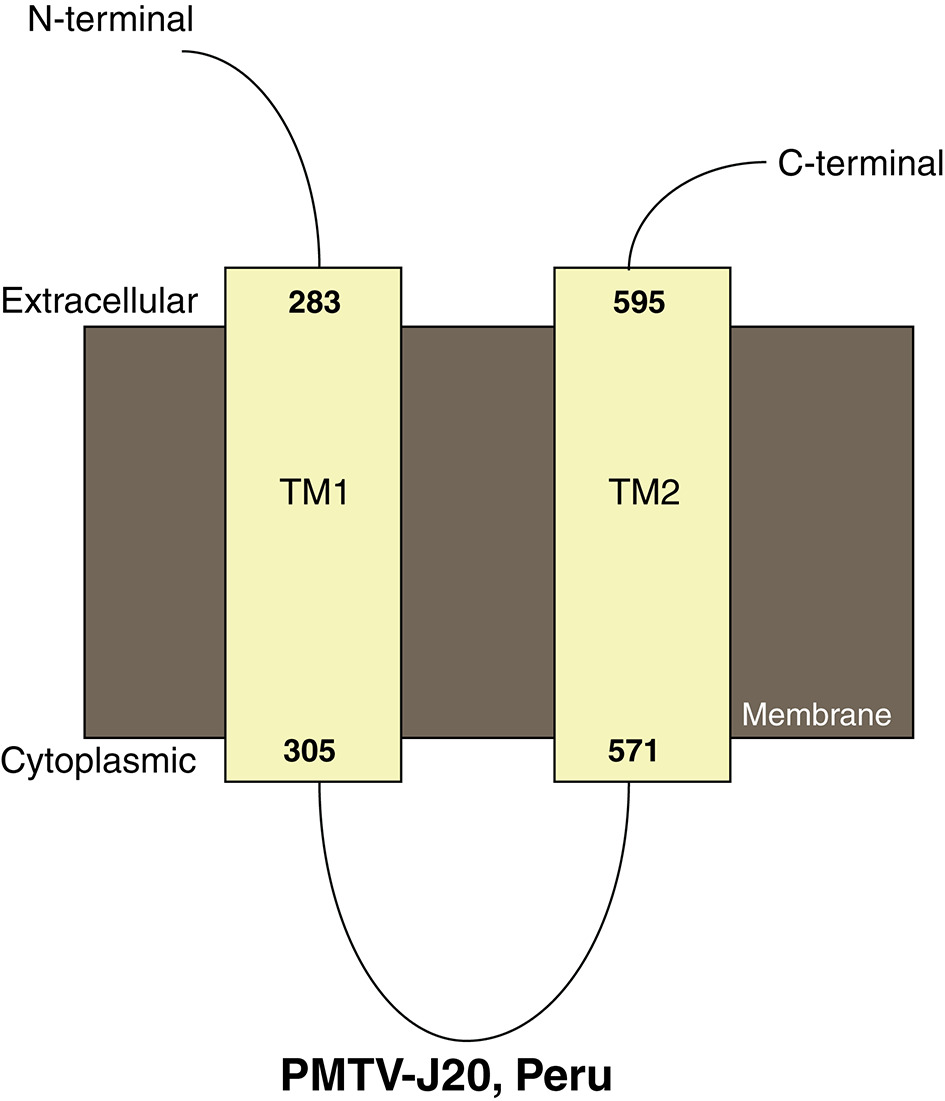

Supplement: Supplementary file 1 — Fig. S1 Predicted transmembrane domains in the CP‐RT region of the J20 isolate using the Phyre2 server. [file MPP-18-864-s001.tif]

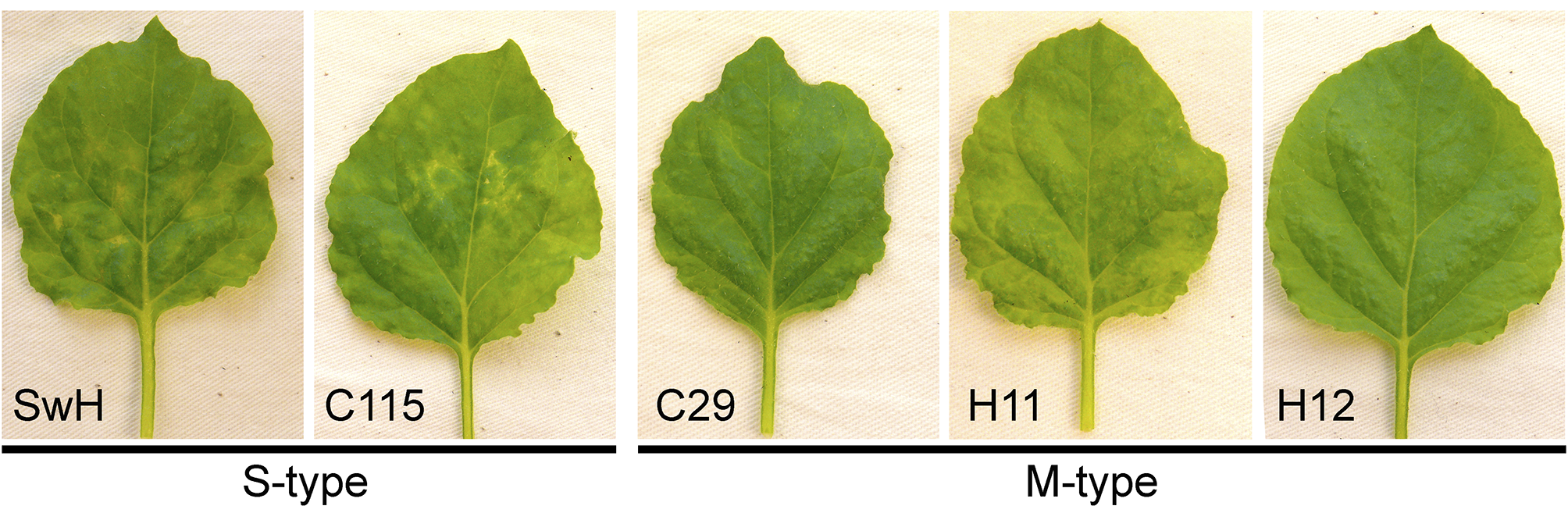

Supplement: Supplementary file 2 — Fig. S2 Symptoms induced in Nicotiana benthamiana by Potato mop‐top virus (PMTV) isolates carrying either severe (S) or mild (M) type of RNA‐CP segment. [file MPP-18-864-s002.tif]
