# Supplementary material for: Molecular and pathobiological characterization of 61 Potato mop‐top virus full‐length cDNAs reveals great variability of the virus in the centre of potato domestication, novel genotypes and evidence for recombination
Source: Mol Plant Pathol. 2017 May 11;18(6):864–77. doi: 10.1111/mpp.12552 (PMC6638219; doi:10.1111/mpp.12552)
Supplement: Supplementary file 3 — Table S1 Geographical locations and GenBank accession numbers of the Peruvian isolates characterized in this study. [file MPP-18-864-s003.docx]

| Isolate name  **Table S1.** Geographic locations and GenBank accession numbers of the newly characterized sequences in this study | Area | District | Date of collection | Geographical  coordinates | Cultivar | Altitude (m) | GenBank accession numbers* |
| --- | --- | --- | --- | --- | --- | --- | --- |
| C29 | Cuzco | Colquepata-Milka | 2009-02-12 | 13°24.517 S 71°39.059 W | Qompis | 3800 | RNA_Rep C29_P69 KU955466  RNA_Rep C29_P70 KU955467  RNA_Rep C29_P71 KU955468  RNA_CP C29_P23 KU955474  RNA_CP C29_P24 KU955475  RNA_CP C29_P96 KU955476  RNA_TGB C29_P40 KU955496 |
| C32 | Cuzco | Colquepata-Chocopia | 2009-02-12 | 13°22.074 S 71°41.589 W | Huayro | 3863 | RNA_CP C32_P25 KU955477  RNA_CP C32_P26 KU955478 |
| C39 | Cuzco | INIA | 2009-02-10 | ? | Andenes | 3560 | RNA_CP C39_P27 KU955479  RNA_CP C39_P28 KU955480  RNA_TGB C39_P9 KU955497 |
| C52 | Cuzco | INIA | 2009-02-10 | ? | Andenes | 3560 | RNA_CP C52_P29 KU955481  RNA_TGB C52_P11 KU955498 |
| C57 | Cuzco | Cuyuni | 2009-02-12 | 13°37.126 S 71°34.760 W | Qompis | 4000 | RNA_CP C57_P31 KU955483  RNA_CP C57_P32 KU955484  RNA_TGB C57_P13 KU955499 |
| C60 | Cuzco | Ccatcca | 2009-02-12 | 13°36.107 S 71°34.467 W | Revolucion | 3763 | RNA_CP C60_P88 KU955485  RNA_CP C60_P89 KU955486  RNA_TGB C60_P15 KU955500 |
| C61 | Cuzco | Ccatcca | 2009-02-12 | 13°36.107 S 71°34.467 W | Cica | 3763 | RNA_Rep C61_P76 KU955469  RNA_Rep C61_P77 KU955470  RNA_CP C61_P33 KU955487  RNA_CP C61_P34 KU955488  RNA_TGB C61_P17 KU955501 |
| C115 | Cuzco | Occopata | 2009-02-13 | 13°36.081 S 71°57.530 W | Qompis | 3995 | RNA_Rep C115_P79 KU955471  RNA_Rep C115_P80 KU955472  RNA_CP C115_P35 KU955489  RNA_CP C115_P36 KU955490  RNA_TGB C115_P20 KU955502 |
| H11 | Huancavelica | Yauli (Sotopampa) | 2009-02-17 | 12°47.0136 S 74°44.7291 W | Qompis | 3775 | RNA_Rep H11_P64 KU955464  RNA_Rep H11_P65 KU955465  RNA_CP H11_P82 KU955491  RNA_TGB H11_P1 KU955503 |
| H12 | Huancavelica | Yauli (Sotopampa) | 2009-02-17 | 12°47.0136 S 74°44.7291 W | Qompis | 3775 | RNA_CP H12_P22 KU955492  RNA_TGB H12_P4 KU955504 |
| J20 | Cajamarca |  | 2011 | ? | ? | ? | RNA_CP J20_J203 KU955493  RNA_TGB J20_P157 KU955505 |
| J21 | Cajamarca |  | 2011 | ? | ? | ? | RNA_CP J21_P106 KU955494  RNA_TGB J21_P117 KU955506 |
| SwH | Götaland, Sweden | Halland | 1999 | ? | Matilda | ? | RNA_Rep KU955473  RNA_CP KU955495  RNA_TGB KU955507 |
|  |  |  |  |  |  |  |  |

* sequences of identical clones (as specified in Table S2) were not submitted the GenBank
