# Supplementary material for: Molecular and pathobiological characterization of 61 Potato mop‐top virus full‐length cDNAs reveals great variability of the virus in the centre of potato domestication, novel genotypes and evidence for recombination
Source: Mol Plant Pathol. 2017 May 11;18(6):864–77. doi: 10.1111/mpp.12552 (PMC6638219; doi:10.1111/mpp.12552)
Supplement: Supplementary file 4 — Table S2 Full‐length cDNA clones (plasmids) of Peruvian isolates of Potato mop‐top virus (PMTV). [file MPP-18-864-s004.doc]

Table S2. Full length cDNA clones (plasmids) of Peruvian isolates of PMTV

| **Isolate** | **RNA-rep** | **RNA-CP** | **RNA-TGB** |
| --- | --- | --- | --- |
| C29 | P69, P70, *P71* | P23*, *P24**, P96 | P40, *P41* |
| C32 | - | P25*, P26 | - |
| C39 | - | P27, P28 | P9, *P10* |
| C52 | - | P29 | P11, *P12* |
| C57 | - | P31, *P32* | P13, *P14* |
| C60 | - | P88*, P89* | P15, *P16* |
| C61 | P76, *P77* | P33, P34 | P17, *P18* |
| C115 | P79, *P80* | P35, *P36* | P20, *P19* |
| H11 | P64, *P65* | P82 | P1, *P2* |
| H12 | - | P22, *P21* | P4, *P3* |
| J20 |  | J20-3, *J20-4, J20-5* | P157, *P160* |
| J21 |  | P106, *P107, P108, P109, P110,* | P117, *P118, P120, P161, P162* |

* shorter version of RNA-CP (internal deletion in the CP-RT cistron)

Italics script signifies identical sequences of the clones for given genome component.
