# Supplementary material for: Molecular and pathobiological characterization of 61 Potato mop‐top virus full‐length cDNAs reveals great variability of the virus in the centre of potato domestication, novel genotypes and evidence for recombination
Source: Mol Plant Pathol. 2017 May 11;18(6):864–77. doi: 10.1111/mpp.12552 (PMC6638219; doi:10.1111/mpp.12552)
Supplement: Supplementary file 5 — Table S3 List of primers used in this study. [file MPP-18-864-s005.docx]

**Table S3:** List of primers used in this study.

| Primer name | Primer sequence (5’-3’) | Purpose |
| --- | --- | --- |
| PMTV-T7-RNA1-F | TAGAGCTC**TAATACGACTCACTATA**GGTATTTTTATCAACTCT | Amplififcation of full-length RNA-Rep |
| PMTV-MluI-RNA-R | AAACGCGTGGTCTTGGATACCCTCCAAGG | Amplification of full-length RNA-Rep and RNA-CP |
| PMTV-T7-RNA-CP-F | AACTGCA**GTAATACGACTCACTATA**GGTATTTTTTAAGTCTAAACAG | Amplification of full-length RNA-CP |
| PMTV-T7-RNA-TGB-F | AAGAGCTC**GTAATACGACTCACTATA**GGTATTTCAACTCTACCTAGCC | Amplification of full-length RNA-TGB |
| SpeI-RNA-TGB-R | AAACTAGTGGTCTTGGATACCCTCCAAGG | Amplification of full-length RNA-TGB |
| RNA1-592-P | CTAAAGTGGTGAGCGCTG | Sequencing of RNA-Rep |
| RNA1-1532-P | GTCGGGTATACTAGAGAG | Sequencing of RNA-Rep |
| RNA1-2161-P | GAGAATACTGAGAATGAG | Sequencing of RNA-Rep |
| RNA1-4326-P | GTTGGCTTTGAACAAGC | Sequencing of RNA-Rep |
| RNA1-4537-P | GAACTCTGAGTGAAATC | Sequencing of RNA-Rep |
| RNA1-4754-N | CCAGCTCGTCAGACGTC | Sequencing of RNA-Rep |
| RNA1 FURO-HEL F | GATGGTGTACCCGGATGTGGAAAGTC | Sequencing of RNA-Rep |
| RNA1-Sal-M-R | CATAAGTCGACTTTCCACATCCGGG | Sequencing of RNA-Rep |
| Kpn-RNA1n-NEW-R | TACTGGGTACCTGGTATTTTCGGT | Sequencing of RNA-Rep |
| EcoRI-RNA1n-R | CATCATGAATTCCAACTAAATCTTG | Sequencing of RNA-Rep |
| PMTV RNA2 del-15-R | GCCGGGGCCCTTAGGACGCGGATCTGATCAC | Sequencing of RNA-CP |
| PMTV RNA2 del-59-R | TCCTGGGCCCTTATGCCTCATCTTTGTTACGA | Sequencing of RNA-CP |
| PMTV RNA2 NheI-F | CTGCTAGCGGCGGCTGCGT | Sequencing of RNA-CP |
| PMTV-J20-678F | GTCCGAGAGAAGCTTCCGC | Sequencing of RNA-CP of isolate J20 |
| PMTV-J20-2200R | GACTCAATAACATCCACAAGTG | Sequencing of RNA-CP of isolate J20 |
| GKT-XhoI-M-R | TCCGCTCGAGGGAACACCAGTCACGGCACC | Sequencing of RNA-TGB |
| R-TGB-765-F | CGGGAAGAGACAGCTCG | Sequencing of RNA-TGB |
| R-TGB-2307-R | CCCATAAGGAAATTCCTG | Sequencing of RNA-TGB |
